# Canoe: An Autonomous Infrastructure-Free Indoor Navigation System

**DOI:** 10.3390/s17050996

**Published:** 2017-04-30

**Authors:** Kai Dong, Wenjia Wu, Haibo Ye, Ming Yang, Zhen Ling, Wei Yu

**Affiliations:** 1 School of Computer Science and Engineering, Southeast University, Nangjing 211189, China; wjwu@seu.edu.cn (W.W.); yangming2002@seu.edu.cn (M.Y.); zhenling@seu.edu.cn (Z.L.); 2 College of Computer Science and Technology, Nanjing University of Aeronautics and Astronautics, Nangjing 210016, China; yhb@nuaa.edu.cn; 3 Department of Computer and Information Sciences, Towson University, Towson, MD 21252, USA; wyu@towson.edu

**Keywords:** IoT, knowledge extraction, indoor navigation, location fingerprinting, missed AP problem, maximum likelihood estimation

## Abstract

The development of the Internet of Things (IoT) has accelerated research in indoor navigation systems, a majority of which rely on adequate wireless signals and sources. Nonetheless, deploying such a system requires periodic site-survey, which is time consuming and labor intensive. To address this issue, in this paper we present *Canoe*, an indoor navigation system that considers shopping mall scenarios. In our system, we do not assume any prior knowledge, such as floor-plan or the shop locations, access point placement or power settings, historical RSS measurements or fingerprints, etc. Instead, Canoe requires only that the shop owners collect and publish RSS values at the entrances of their shops and can direct a consumer to any of these shops by comparing the observed RSS values. The locations of the consumers and the shops are estimated using maximum likelihood estimation. In doing this, the direction of the target shop relative to the current orientation of the consumer can be precisely computed, such that the direction that a consumer should move can be determined. We have conducted extensive simulations using a real-world dataset. Our experiments in a real shopping mall demonstrate that if 50% of the shops publish their RSS values, Canoe can precisely navigate a consumer within 30 s, with an error rate below 9%.

## 1. Introduction

The Internet of Things (IoT) is considered to be the next generation in industrial evolution [1]. With a wealth of potential applications, indoor navigation systems are of particular interest. There are many scenarios, in which location-based services are useful. For instance, a consumer may want to find a certain shop in a shopping mall to optimize or enhance their experience, while shop owners want to direct consumers to their shops in order to increase business. This implies a strong need for a navigation service, which is capable of determining a target’s location relative to a user’s current location.

With the development of IoT, navigation services can be implemented based on a localization system, which relies on users’ mobile devices and adequate wireless signals and sources in the environment, in performing vector subtraction between two absolute locations. Notice that a Wi-Fi-based location fingerprinting (LF) technique is “infrastructure-free”: it can provide value-added localization services for existing WLAN infrastructures without the need for any specialized hardware [2]. Nonetheless, deploying a traditional LF-based indoor positioning system is not easy, since the positioning server should collect the location fingerprints by performing a site-survey of the received signal strength (RSS) from multiple access points (APs). With these fingerprints, the positioning server is able to localize a mobile device based on its RSS measurements. Nonetheless, the site-survey is extremely time consuming and labor intensive, which raises the cost of initiating an LF-based localization service.

Furthermore, the positioning server should perform the site-survey periodically to update the fingerprints, reducing errors caused by the changing Wi-Fi environment, but also raising the cost of maintaining the service. Following [3], a number of research studies has been performed to enable the collection of fingerprints based on crowd-sourced solutions instead of an off-line site-survey. In these solutions, users are required to continuously upload RSS measurements to the positioning server as training data. Nonetheless, this can raise significant privacy concerns, and incentive mechanisms need to be in place to guarantee the number of voluntary participants.

It is worth noting that the aforementioned crowd-sourcing approaches are easy to deploy, while still enabling localization with high accuracy. Nonetheless, there is still a gap between “easy to deploy” and “no need to deploy”. If there is no deployed localization service in a shopping mall, it is difficult for a consumer to acquire his/her location. In this paper, we introduce an autonomous navigation solution without a dedicated localization service or infrastructure deployed. Our designed solution only utilizes the published RSS values at shop entrances for navigational computation. Notice that in our design, we assume that a shop owner is willing to direct consumers to his/her shop by publishing the RSS measurements observed at his/her shop entrance, and we also assume that a consumer can easily acquire the RSS measurements at his/her current location using his/her mobile device. In addition, we do not assume other knowledge, including the floor-plan, the AP placement, power settings, historical RSS measurements or fingerprints.

In any indoor environment, like that of a shopping mall, the accessible regions for personal traffic are limited by walls and indoor infrastructure, and individuals can only walk in nearly straight paths through the hallways [4]. Therefore, we simplify the indoor navigation to an either-or operation, i.e., “Should the consumer move forward or backward?”. Our basic idea is to determine whether a consumer is approaching or leaving a target shop, by computing the distance between the RSS measurements at the consumer’s current location and those at the shop’s entrance. With all of the acquired RSS observations, including those collected by the consumer’s mobile device as the user travels and those published at the target location, where each can be represented as a point in a same *m*-dimensional signal space (where *m* is equal to the number of the APs), the physical distance between the locations of different RSS observations can be computed based on the *Euclidean* distance of these observations in the signal space. Then, what is left is a simple decision: if the distance between the consumer’s latest RSS observation and that of the target location is decreasing, the consumer should move forward (i.e., continue in the same direction); otherwise, he/she should move backward.

Nonetheless, it is quite challenging to precisely estimate the distance between RSS observations. The main problem concerns the dimensionality mismatches in the signal space. This is because various RSS observations can observe different APs. In the case where the consumer is far from the target shop, it is likely no APs will cover both locations. Even if two observing locations are close to each other, the observed APs can still be significantly different, due to the missed AP phenomenon. Studies on Wi-Fi fingerprinting approaches in [5,6,7] have shown that when a mobile device performs a Wi-Fi scan, some of the APs can be missed in a scanning cycle, causing dimensionality mismatches between different RSS observations. In addition, if the Wi-Fi scan operation is performed frequently (e.g., every three seconds), the number of missed APs will be greatly increased. Furthermore, even if a subset of RSS observations that share a few common APs can be placed into a signal space with reduced dimensions, distance estimation based on insufficient information will often lead to significant errors when making the “forward” or “backward” decisions.

To address these challenges, in this paper, we propose a novel algorithm that computes the RSS values of missed APs from the values of those observed, using the maximum likelihood estimation (MLE). Our designed algorithm begins with assigning arbitrary initial values to the unknown parameters, including the locations of the consumer, target shops, APs and the AP’s power settings. Then, a stochastic gradient descent optimization technique is designed to iteratively learn the values of these parameters to maximize the likelihood of observing the RSS measurements with respect to the estimated locations of the consumer, the shops and the APs, under the assumption that the RSS measurements (in dBm) are distributed through Gaussian distribution and that the path loss varies exponentially with distance. Finally, each of the RSS observations is represented as a point in a linear signal space, without reducing dimensionality. In doing this, navigation errors can be greatly reduced, as every one of the RSS measurements can contribute to the navigation, rather than only a small portion of the measurements contributing.

Designing such an MLE-based algorithm is non-trivial, as there are many parameters to consider for each RSS observation. As a result, overfitting can easily occur (i.e., the learned parameters deviate to describe the random noises of the RSS measurements, instead of the underlying physical relationship between the observing locations and those of the APs). Lacking sufficient RSS observations to control the extent of regularizing the learned values via techniques like cross-validation, we instead use an early stopping strategy to terminate the fitting process. In designing our strategy and learning algorithm, we utilize simulations based on a public dataset and implement a prototype application. In addition, to further evaluate the effectiveness of Canoe, we have conducted live experiments in a real shopping mall. The experimental results show that, if 50 % of the shops publish their RSS measurements, Canoe can direct a consumer to any of these shops after nine RSS observations (i.e., 30 s) with an error rate of only 9 %.

The remainder of this paper is organized as follows. In Section 2, we first describe a motivating scenario and introduce the primary challenge and solution. In Section 3, we present the estimation model and learning algorithm used in Canoe. In Section 4, we use a simulation to learn the optimal settings of Canoe and address the overfitting problem. In Section 5, we show live experiments in a real shopping mall to evaluate the effectiveness of Canoe. We conduct a literature review in Section 6, followed by conclusions in Section 7.

## 2. Problem Definition

In this section, we first introduce a motivated scenario, and then present the problem definition and our solution. In Table 1, we summarize the main notations introduced throughout this article.

### 2.1. Motivated Scenario

Shop owners are always willing to attract more consumers to increase sales through advertising, typically providing store information and sales and the corresponding locations. This is even more likely in a shopping mall, in which there are typically competing shops selling similar or even the same commodities. Consumers may also hope to know the locations of these shops. For example, the consumer, who is looking for the nearest Starbucks to have a cup of coffee and take a break, does not want to spend time wandering around the mall.

Both the shop owners and the consumers have a strong need for an indoor navigation service. Nonetheless, there is no such service available in most shopping malls. The main reason for this is that neither a shop owner nor a consumer can deploy such a service based on the existing positioning techniques, as these techniques generally rely on the maintenance of a large amount of dynamic location fingerprints and knowledge of the whole floor plan.

The owner of the mall may have the means, but may also have scant motivation to deploy such an in-building navigation service, since the owner cares more about whether a user is shopping in his or her mall rather than at whichever specific shops. As an example, Google’s Indoor Maps provides localization and navigation services based on publicly broadcast data (e.g., Global Positioning System (GPS), Wi-Fi and cell tower information), and they are currently available only in selected locations, listed in [8] (i.e., unavailable in all other locations). To make Indoor Maps available inside a building, the owner should upload a floor plan and periodically perform a site-survey by walking through the whole building to improve location accuracy, which is quite time consuming and labor intensive. This leads to reluctance to adopt and deploy the service.

For navigation in the aforementioned scenario, we introduce Canoe. It is a lightweight navigation system with no prior deployment of dedicated localization infrastructure, which requires only minor efforts of the shop owners and the consumer. We assume that the shop owners (here denoted “shop-users”) are willing to publish the RSS measurements observed at the entrance of their shops. We do not assume the prior knowledge of the locations of these shops, a floor plan, AP placement and power settings, or any other information, which is not accessible to the shop-users and customers (here denoted “consumer-users”).

With Canoe, a consumer-user with a smart phone continuously observes his/her current RSS measurements to compare to those published by the target shop, so that he or she can “paddle his/her own Canoe” to direct him/herself to the target shop, without disclosing his or her information and without requiring cooperation of any other consumers.

### 2.2. Problem Definition and Solution Overview

We consider the indoor navigation scenario based on the motivated scenario described in Section 2.1, where the shop-users and the consumer-user observe the RSS measurements at their own locations from *m* APs with unknown locations, denoted as:

(1) $$A=\{A_{1},A_{2},\ldots ,A_{m}\}.$$

Thus, an RSS observation can be represented as an *m*-dimensional vector:

(2) $$I=<I_{A_{1}},I_{A_{2}},\ldots ,I_{A_{m}}>,$$

where the value in each dimension represents the RSS measurement from a unique AP. For the APs that cannot be observed in an RSS observation (e.g., when the APs are far from the observing location), we tag the values in the corresponding dimensions as “0”.

The shop-users each continuously observe the RSS measurements at the entrance of their own shop and periodically publishes the averaged values over a given time interval. We use an $m\times n_{s}$ matrix $I^{\prime }$ to denote the averaged RSS measurements, where $n_{s}$ is the number of shops.

$$I^{\prime }=\left[I_{1}^{\prime },I_{2}^{\prime },...,I_{n_{s}}^{\prime }\right]=\left[\begin{array}{ccc} I{}_{1,A_{1}}^{\prime } & \cdots & I{}_{n_{s},A_{1}}^{\prime }\\ \vdots & \vdots & \vdots \\ I{}_{1,A_{m}}^{\prime } & \ldots & I{}_{n_{s},A_{m}}^{\prime } \end{array}\right]$$

As the consumer-user moves in an arbitrary direction, his or her mobile device obtains $n_{c}$ observations of the RSS measurements. We use an $m\times n_{c}$ matrix $I^{\prime \prime }$ to denote the observed RSS measurements.

$$I^{\prime \prime }=\left[I_{1}^{\prime \prime },I_{2}^{\prime \prime },...,I_{n_{c}}^{\prime \prime }\right]=\left[\begin{array}{ccc} I^{\prime }{}_{1,A_{1}}^{\prime } & \cdots & I^{\prime }{}_{n_{c},A_{1}}^{\prime }\\ \vdots & \vdots & \vdots \\ I^{\prime }{}_{1,A_{m}}^{\prime } & \ldots & I^{\prime }{}_{n_{c},A_{m}}^{\prime } \end{array}\right]$$

At each observing location, only the nearby APs can be observed. This means that a large proportion of the elements in $I^{\prime }$ and $I^{\prime \prime }$ are tagged as “0”. In addition, when the Wi-Fi scan operations are performed frequently, many more APs can be missed in the RSS observations. As a result, both $I^{\prime }$ and $I^{\prime \prime }$ are sparse matrices.

On a certain floor in a shopping mall, where the accessible region is limited and the path-finding result to a shop can be relatively simple, Canoe can tell whether the user is approaching or leaving the target shop based on the distance between the user’s current location and destination. Therefore, we simplify the navigation problem to a distance estimation problem, which is defined as follows:

**Problem** **1.** Given $I^{\prime }$ and $I^{\prime \prime }$, for an arbitrary element $I_{i}^{\prime }\in I^{\prime }$ as the target, and for each $I_{j}^{\prime \prime }\in I^{\prime \prime }$, how do we compute the distance between the locations $L_{I_{i}^{\prime }}$ and $L_{I_{j}^{\prime \prime }}$? Here, $L_{I_{i}^{\prime }}$ and $L_{I_{j}^{\prime \prime }}$ respectively represent the locations, where $I_{i}^{\prime }$ and $I_{j}^{\prime \prime }$ are observed.

The physical distance between two observing locations can be estimated from the Euclidean distance between the RSS observations in the signal space [9,10]. Nonetheless, since $I^{\prime }$ and $I^{\prime \prime }$ are sparse matrices, it is always difficult to compute the Euclidean distance between two elements $I_{i}^{\prime }\in I^{\prime }$ and $I_{j}^{\prime \prime }\in I^{\prime \prime }$ due to dimension mismatches. Canoe addresses this problem by estimating all of the unobserved RSS values from all of the APs. This estimation is based on the theory that various RSS measurements are not independent of each other, but are strongly correlated. Although the locations of the observations and the APs are unknown, the correlations between these locations really determine the RSS measurements. In other words, the observed RSS measurements may reflect the relative locations of the observations and the APs, so that they can contribute to estimates of the unobserved values.

In the following, we present the design detail of Canoe.

### 2.3. Design Overview

Notice that it is quite challenging to precisely estimate the distance between RSS observations, concerning the dimensionality mismatches in the signal space as various RSS observations can observe different APs. On the one hand, if the consumer is far from the target shop, it is likely no APs will cover both locations. On the other hand, if two observing locations are close to each other, the observed APs can be significantly different because of the missed AP phenomenon. In addition, if a subset of RSS observations that share a few common APs can be placed into a signal space with reduced dimensions, distance estimation based on insufficient information will often lead to significant errors.

**Figure 1:**
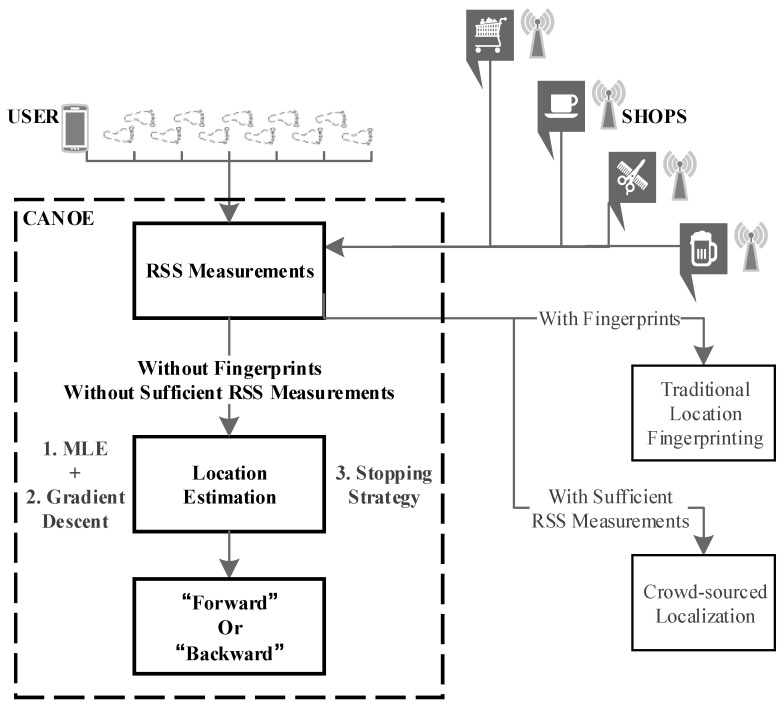
Canoe overview.

To address these issues, our designed system consists of three key components: (i) the estimation model, (ii) a learning algorithm and (iii) a mechanism to deal with overfitting. Figure 1 illustrates how Canoe navigates a consumer to an arbitrary shop. When an offline fingerprint database is available, traditional location fingerprinting techniques can be used to accurately locate the users and the shops; or when there are sufficient online RSS measurements, crowd-sourced localization techniques can be used to generate fingerprints. In the case that no fingerprint database and only insufficient RSS measurements are available, Canoe can still perform the navigation.

On the hypothesis that the RSS measurements (in dBm) are normally distributed and that the path loss varies exponentially with distance, the relative likelihood of observing an RSS measurement can be described by a probability density function. Canoe uses the maximum likelihood estimation to maximize this likelihood (with respect to minimizing the distances between the computed values and the real values of the observed RSS measurements). In doing this, Canoe can tell whether a user should move forward or backward based on the estimated locations.

## 3. Design Detail of Canoe

In this section, we present the design detail of Canoe. In the following, we first overview the key components and then present the individual components in detail.

### 3.1. Overview of Key Components

First, related to estimation model, we propose a novel algorithm based on maximum likelihood estimation.

Second, regarding the learning algorithm, we adopt a stochastic gradient descent optimization technique, which is designed to iteratively learn the values of these parameters to maximize the likelihood of observing the RSS measurements with respect to the estimated locations of the consumer, the shops and the APs. Thus, combining the maximum likelihood estimation and stochastic gradient descent optimization techniques, we solve the dimension mismatch problem, leading to the accurate estimation of the locations of the shops and the consumers.

Third, as there are many parameters to consider for each RSS observation, overfitting can easily occur as a consequence. This means that the learned parameters could deviate to describe the random noises of the RSS measurements, instead of the underlying physical relationship between the observing locations and those of the APs. To overcome this issue, we design an early stopping scheme so that the iteration process is curtailed when the learned results best describe the real locations of observations.

In the following, we introduce these key components in our systems in detail.

### 3.2. Estimation Model

We now introduce the estimation model in detail. We begin with introducing assumptions and then present the estimation model in Canoe. Notice that we use calligraphic uppercase letters to denote matrices and normal uppercase letters with subscripts to denote the elements of the matrices.

We take the following two assumptions in designing Canoe: (i) the Wi-Fi RSS distribution, and (ii) the Wi-Fi signal propagation path loss.

RSS distribution: One common assumption is that the RSS measurements (in dBm) are distributed by the Gaussian distribution [2] described below, while some research has reported that this may not be always the case [11]. Nonetheless, in the literature, it is hard to tell which function could best approximate the real distribution of RSS measurements. Thus, we have chosen to use the most widely-used assumption.

**Assumption 1** (RSS distribution). *The RSS measurements (in dBm) are distributed by following the Gaussian distribution:*

(3) $$I∼Gaussian(\tilde{I},\sigma^{2}).$$

Signal propagation path loss model: The path loss (PL) that a signal encounters inside a building can be predicted by a radio propagation model.

**Assumption 2** (Signal propagation path loss). *During the Wi-Fi signal propagation, the path loss varies exponentially with distance,*

(4) $$PL=PL_{0}+10n\log_{10}\frac{d}{d_{0}}+X_{g}.$$

Here, $PL_{0}$ is the path loss at the reference distance $d_{0}$, *n* is the propagation path loss exponent, *d* is the length of the path, $d_{0}$ is the reference distance (e.g., $d_{0}=1\;m$), and $X_{g}$ is a normal (or Gaussian) random variable with zero mean.

For the sake of convenience, we use $I_{0,j}$ to represent the RSS measurement from the *j*-th AP at $d_{0}=1\;m$ of distance; we have:

(5) $$I_{i,j}=-10n\mathrm{lg}D_{i,j}+I_{0,j},$$

where $I=I^{\prime }\cup I^{\prime \prime }$ is the set of all accessible RSS measurements and $D_{i,j}$ represents the physical distance between the locations of the *i*-th observation and the *j*-th AP.

**Figure 2:**
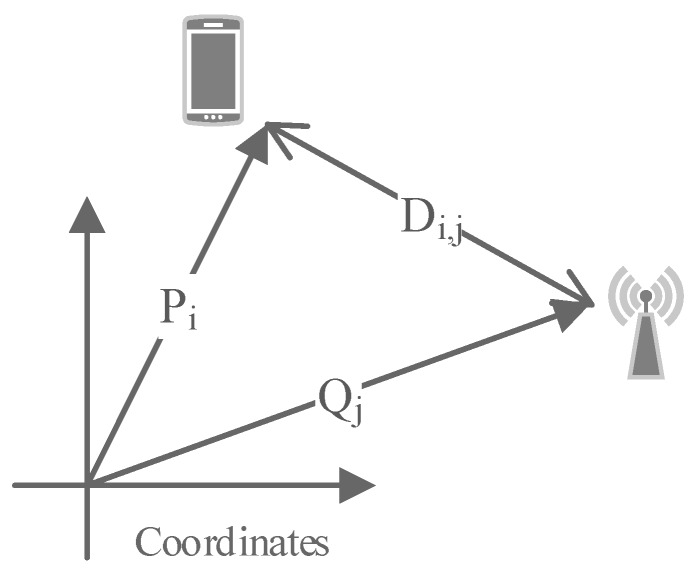
The relations among the location of the observation (denoted as $P_{i}$), the location of the AP (represented as $Q_{j}$) and the distance between the two locations (denoted as $D_{i,j}$).

In Canoe, we use the maximum likelihood estimation to estimate locations. Suppose there is a unified coordinate system to specify all of the locations on the given floor in the shopping mall. For any RSS measurement $I_{i,j}$ in I, we use $P_{i}$ to denote the coordinates of the location of the $i_{th}$ observation and $Q_{j}$ to denote the coordinates of the location of the *j*-th AP. Then, the distance between the two locations $D_{i,j}$ is relevant to $P_{i}$ and $Q_{j}$, as shown in Figure 2. Then, we have:

(6) $$D_{i,j}={∥P_{i}-Q_{j}∥}_{2}.$$

From the set of all accessible RSS measurements I, it is possible to compute the distance $D_{i,j}$ for each observation and for each observed AP. We define the process of location estimation, which estimates the locations of the observations and the APs (matrices P and Q) from the distances (matrix D).

**Definition 1** (Location estimation). A set of RSS measurements in dBm is represented as $I\in R^{{-}^{m\times n}}$, find $P\in R^{{+}^{m\times 2}}$ (or $R^{{+}^{m\times 3}}$ in 3D positioning) and $Q\in R^{{+}^{m\times 2}}$ (or $R^{{+}^{m\times 3}}$), such that a divergence function $F(I||\tilde{I})$ is minimized, where ${}^{a)}$$\tilde{I}$ is the matrix transformed from $\tilde{D}$ by Assumption 2; and ${}^{b)}$$\tilde{D}\in R^{{}^{m\times n}}$ is the reconstructed matrix by Equation (6).

The objective function is generated as follows. When we take Assumption 1, we have the probability density function,

(7) $$f_{I}\left(I_{i,j}\right)=\frac{1}{\sqrt{2\pi }\sigma }\exp\left\{-\frac{{\left(I_{i,j}-{\tilde{I}}_{i,j}\right)}^{2}}{2\sigma^{2}}\right\}.$$

Maximizing the likelihood of observing I, with respect to P, Q and $I_{0}$ under the i.i.d. (independent and identically distribution) assumption,

(8) $$L\left(P,Q,I_{0}|I\right)=\prod_{i,j}f_{I}\left(I_{i,j}\right),$$

is equivalent to minimizing:

(9) $$\begin{array}{cc} F(I||\tilde{I}) & =\sum_{i,j}{\left(I_{i,j}-{\tilde{I}}_{i,j}\right)}^{2} \end{array}$$

(10) $$\begin{array}{c} =\sum_{i,j}{\left(I_{i,j}-I_{0,j}+10nlg{∥P_{i}-Q_{j}∥}_{2}\right)}^{2} \end{array}$$

Based on this objective function, Canoe learns the location estimation model by fitting the previously observed RSS measurements in I.

### 3.3. Learning Algorithm

In an indoor navigation application, which directs consumers to their destinations, real-time feedback is essential to correcting the walking directions. Because the stochastic gradient descent optimization combines implementation ease with a relatively fast running time, we choose it as the learning algorithm in Canoe to minimize the objective function in Equation (10).

In particular, Canoe loops through all of the observed RSS measurements in I. For each RSS measurement, Canoe estimates ${\tilde{I}}_{i,j}$ and computes the associated estimation error:

$$\begin{array}{cc} E_{i,j} & =I_{i,j}-{\tilde{I}}_{i,j}=\left(I_{i,j}-I_{0,j}+10nlg{∥P_{i}-Q_{j}∥}_{2}\right). \end{array}$$

Then, Canoe modifies the parameters in the opposite direction of the gradient, yielding:

(11) $$\begin{array}{cc} P_{i} & P_{i}-\alpha \cdot \frac{\partial F}{\partial P}=P_{i}-2\alpha \cdot \frac{10n\cdot \left(P_{i}-Q_{j}\right)E_{i,j}}{\ln10\cdot {∥P_{i}-Q_{j}∥}_{2}} \end{array}$$

(12) $$\begin{array}{cc} Q_{j} & Q_{j}-\alpha \cdot \frac{\partial F}{\partial Q}=Q_{j}+2\alpha \cdot \frac{10n\cdot \left(P_{i}-Q_{j}\right)E_{i,j}}{\ln10\cdot {∥P_{i}-Q_{j}∥}_{2}} \end{array}$$

(13) $$\begin{array}{cc} I_{0,j} & I_{0,j}-\alpha \cdot \frac{\partial F}{\partial I_{0}}=I_{0,j}+2\alpha E_{i,j} \end{array}$$

where α is the step size and *n* is the propagation path loss exponent. Notice that the value of *n* can be various in different indoor environments and is normally in the range of two to four. Fortunately, we can fix *n* to any positive value. In our simulation and experiments, we find that no matter which value we set to *n*, the localization results remain the same. This is because Canoe only computes the relative locations in a random coordinate system, and with different values of *n*, the output locations of Canoe differ with each other just as if they belong to different coordinate systems. Canoe neither requires the knowledge on which coordinate system is used, nor requires references to cardinal directions. It only requires the information of relative distance between locations so as to perform the “forward or backward” navigation.

At convergence, in theory, the locations of all of the observations and the APs (and also the power settings of the APs) can be estimated. These locations give expression to the physical distances/relationship between each other (i.e., the distribution of these locations), which maximizes the likelihood of observing the known RSS measurements. Nonetheless, in practice, the results at convergence can still deviate far from the actual locations, even if the coordinate system is converted carefully through a stretch, spin or flip operation in the whole area. This is because the results can be highly prone to overfitting.

### 3.4. Overfitting Issue and Solution

There are a number of parameters (i.e., all elements in matrices P, Q and $I_{0}$) relative to the number of observed RSS measurements (i.e., matrix I) in location estimation. Thus, overfitting can easily occur in the iterations performed in Equations (11), (12) and (13).

To avoid overfitting in the stochastic gradient descent, one typical solution is to conduct regularization. Nonetheless, the regularization is essentially a feature selection by penalizing complex models to produce sparse feature matrices, while in Canoe, all of the features (i.e., P, Q and $I_{0}$) are indeed irreducible. This is because these features will affect the values of the RSS measurements in I in the physical world, and the model in Canoe is fixed if Assumptions 1 and 2 are taken. In addition, the lack of ground truth makes it difficult, if not impossible, to perform a regularization.

Due to these limitations, Canoe uses a stopping strategy rather than normal regularization to avoid overfitting. Via simulations, we find that, in the iterative process, the fitting results (i.e., the estimated locations of the consumer and the shops in P) quickly follows a similar location distribution with that of the real locations, before the fitting results start to describe the random noises of the RSS measurements, and the parameters are learned to be at increasingly erroneous locations. The challenge is to determine when to stop the iteration. We describe in detail the early stopping strategy in Section 4.4.

## 4. Implementation and Simulation Study

In this section, we introduce the implementation and simulation study in detail. In the following, we first give the overview and dataset. We then introduce simulation results related to missed APs and the failure rate, the impact of overfitting and the effectiveness of our designed strategy, as well as the simulation results.

### 4.1. Overview

Recall that the goal of the Canoe system is to provide indoor navigation in cases where no prior knowledge of the building layout or infrastructure is required. It is not designed for any particular building, but instead should provide a general solution for most in-building shopping or other similar scenarios. To implement Canoe, we have simulated a shopping scenario based on a public dataset of Wi-Fi RSS measurements. Based on the simulation results, we have evaluated the best settings of parameters in Canoe and designed an early stopping strategy to avoid overfitting. Then, we have implemented a prototype navigation application and conducted real-world experiments in an actual shopping mall, which will be introduced in Section 5.

### 4.2. Dataset

The public dataset that we use is the mannheim/compass dataset [12]. This is the only public dataset that we could find with recorded traces of signal strength of 802.11 APs. The dataset contains data in both an off-line training phase and an on-line positioning phase, in an area of about 15 m in width and 36 m in length. The off-line fingerprinting data contains 146,080 measurement records for 166 locations (880 records each), and the on-line positioning data contains 6600 measurement records for 60 locations (110 records each).

### 4.3. Missed APs and Failure Rate

**Figure 3:**
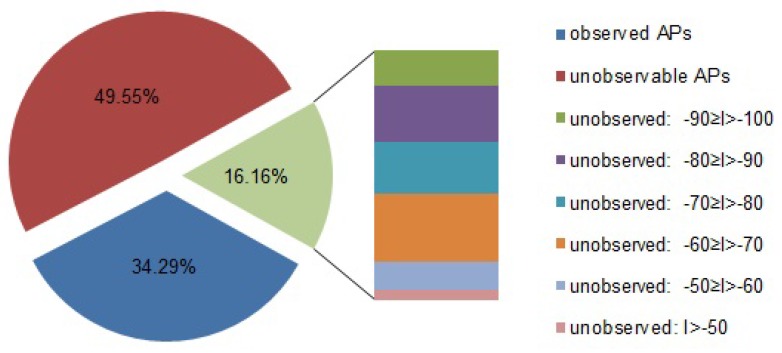
Proportion of the observed APs, the unobservable APs and the missed APs. Here, *I* is referred to as the theoretical RSS, which is an average value from the records in which this AP is observed.

There are in total 20 APs in the dataset. For a given RSS measurement record, these 20 APs can be classified into the following three categories:

The proportions of the observed APs, the unobservable APs, and the missed APs are shown in Figure 3. One interesting finding is that the probability of missing an AP is not obviously related to the averaged RSS value *I*.

- Observed APs: Those are observed in the RSS record (of one-time observation). In contrast, the unobserved APs are those that are not observed in the record.
- Unobservable APs: Those are ones that cannot be observed at the observing location. An AP is considered to be unobservable if no records at this location ever observed this AP. Notice that the unobservable APs must be unobserved APs, but unobserved APs may be observable in different observations at the same location.
- Missed APs: Those are observable, but unobserved in this record. Because some records at this location observe this AP, we can compute an averaged RSS value *I*.

In the dataset, a record can observe seven (20×34.29%) APs on average. This inadequate number of observed APs will always result in navigation failures (i.e., no results can be computed regardless of accuracy or precision). The reason is that different APs are observed in different records at different locations. Only if at least three common APs are shared between four observations, it is possible to compute the geographical relation between these observing locations. Thus, if we only use the observed values for navigation, the failure rate can be very high. In Canoe, since the RSS values from missed APs and unobservable APs can be estimated, the failure rate is always zero. It is worth mentioning that the failure rate increases in the real shopping mall scenario via our real-world experiments. We will discuss this problem in Section 5.

### 4.4. Overfitting and Stopping Strategy

**Figure 4:**
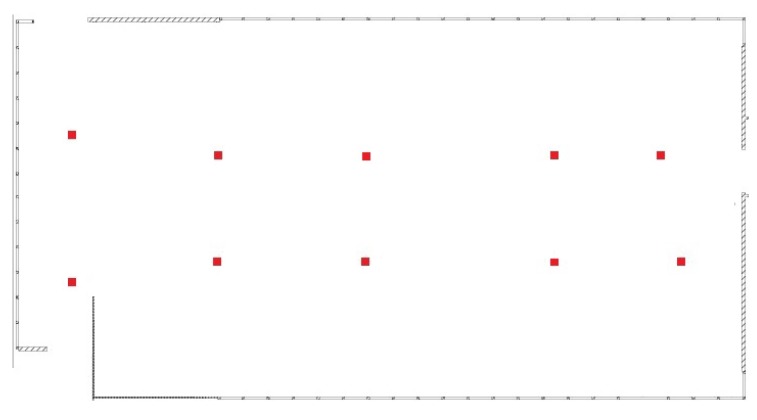
Ten selected locations we used in our case study in Section 4.4.

**Figure 5:**
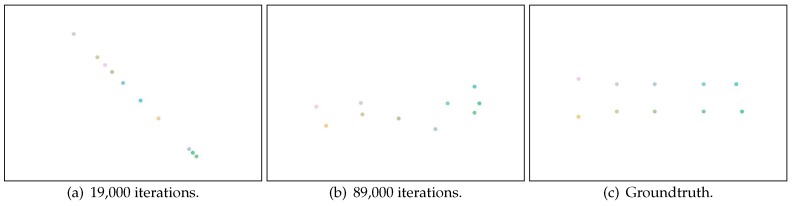
Stopping strategy: (**a**) learned locations with stopping strategy (after the first fluctuation); (**b**) the final result, which is the learned locations with stopping strategy (after the second fluctuation); (**c**) the ground truth.

As we discussed in Section 3.4, overfitting is a key challenge in implementing Canoe, since there are a number of parameters relative to observed RSS measurements. We use a case study to illustrate how the overfitting problem can affect the estimation results and explain how we solve this problem in Canoe. In this case study, we select 10 locations (represented as red dots in Figure 4), and for each location, we randomly select 10 observations of RSS measurements. Using only these data, we learn the locations of the observations using Canoe and show the results after increasing numbers of iterations. We use a unique color for each observing location, and the ground truth is shown in Figure 5c.

**Figure 6:**
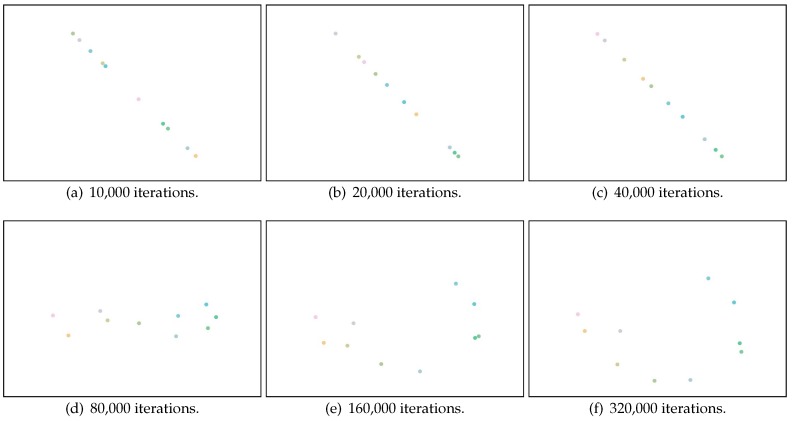
Learned locations after the number of iterations.

Figure 6a–f illustrates the learned locations after 10,000 to 320,000 iterations. It is obvious that the results after approximately 80,000 iterations are the most accurate. After that, overfitting occurs, and the learned locations start to describe the random noise in the RSS observations. This results in great errors in learning the locations at convergence after about 320,000 iterations.

Thus, a stopping strategy should be in place to avoid overfitting. If the relations between the real locations of the shops are unknown, it is difficult to stop the iterations based on the learned values. Furthermore, even if the consumer’s moving pattern can be obtained by using his or her smart phone sensors (i.e., when the relations between the consumer’s observing locations are known), it is still difficult to stop the iteration process at exactly the time when the learned results have the best performance. This is because the RSS measurements are unstable in real Wi-Fi environments. Noise in the RSS measurements observed by the consumer will cause the iteration process to end at a very early stage.

**Figure 7:**
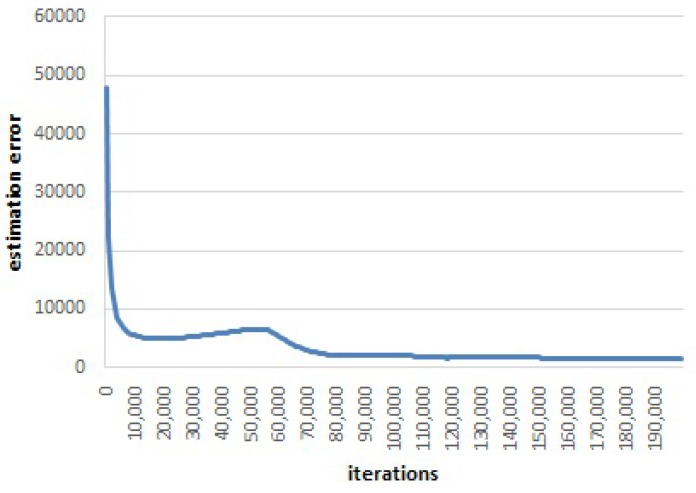
Estimation error versus the number of iterations.

**Figure 8:**
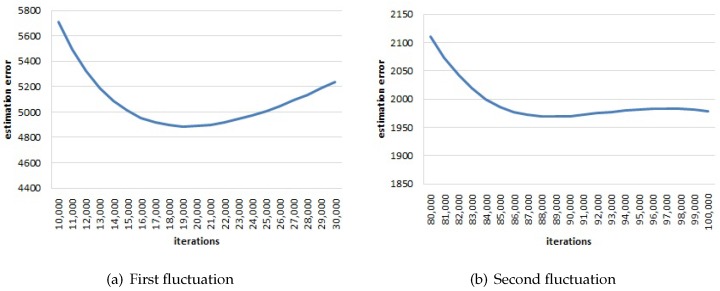
(**a**) The first fluctuation: the estimation error increases after about 19,000 iterations. (**b**) The second fluctuation: the estimation error increases after about 89,000 iterations.

In Canoe, we adopt a strategy to stop the iteration process, which is not based on the learned locations, but is based on the variation of the estimation error $F(I||\tilde{I})$ in Equation (9). When the value of $F(I||\tilde{I})$ decreases with iterations, it also presents violent and frequent fluctuations. We use sliding windows to compute the averaged estimation error. Figure 7 and Figure 8 illustrate the average estimation error versus the number of iterations when the window size is 1000 iterations. There are three fluctuations in total.

The averaged estimation error first decreases rapidly. Then, after around 19,000 iterations, the error increases and then decreases (as shown in Figure 8a). Furthermore, after about 89,000 iterations, the error increases again and then decreases to convergence (as shown in Figure 8b). The estimated results (learned locations) at each fluctuation (i.e., after 19,000 iterations and 89,000 iterations) are shown in Figure 5a and Figure 5b. When the estimation error first increases, the learned locations are distributed in a line and start to describe the 2D relations between each other. When the estimation error increases the second time, the learned locations are distributed in a 2D space and start to describe the random noise in the RSS observations. From this finding, we design the following stopping strategy: when the averaged estimation error increases the second time, Canoe should stop the iteration process.

### 4.5. Simulation and Results

Using the mannheim/compass dataset, we generate simulated scenarios and evaluate the error rate in navigation. In the simulation, we generate RSS measurements observed by the shop-users and by the consumer-users. We assume the rooms to be different shops and randomly select $n_{s}=10$ shops as the shop-users of Canoe.

From these shops, we randomly select one shop as the target shop. For each shop, the closest off-line location to the door is treated as the entrance. At each entrance, we randomly select 10 records and compute the averaged values. These values compose the matrix $I^{\prime }$. Then, we generate consumer-user traces from the off-line locations. To generate a trace, we first randomly select a start location and a direction (north, south, west or east). For every step at a fixed distance d=3m on the direction (Different mobile devices can perform the Wi-Fi scans at different frequencies. The highest frequency for most devices is about three seconds. The walking speed is about 1 m/s. Thus, we have d=3m.), we find the closest off-line location and randomly select one record as the RSS measurements observed by the consumer. After $n_{c}$ steps, we can have $n_{c}$ RSS records in total, which compose the matrix $I^{\prime \prime }$. Based on the data (matrix $I^{\prime }$ and $I^{\prime \prime }$), Canoe determines whether the consumer is approaching or leaving the target shop (or whether the consumer should move forward or backward).

After Canoe has its answer, we compare it with the correct answer, which is computed from the distance between the consumer’s locations and the shops’ locations. Notice that in the simulation, the distance between the consumer’s location and the target shop’s location is set to be over five meters. When the two locations are closer to each other, it is difficult to estimate whether the consumer is approaching or leaving the shop.

We repeat this simulation and compute the mean error rate. The results are shown in Table 2. As we can see from the table, using only two observations, the navigation results are only 75 % correct. If the consumer continues moving in his/her direction and collects five observations, 90 % of the navigation results are correct.

## 5. Real-World Experiments

We have conducted real-world experiments in a real shopping mall to evaluate the feasibility of Canoe in real-world practice. The shopping mall that we chose is a representative indoor environment for navigation.

First, this shopping mall has in total two buildings in operation (and two other buildings under construction). This is a very large shopping area, so that consumers in this mall may be confused when finding a certain shop, especially when this shop is new or recently moved to a new location in the mall. The experiments are performed on Level B-1, which is the only floor connecting both buildings. The floor-plans of both buildings are available at [13], by switching between buildings on top right of the page. To check the floor-plan of Level B-1, one should choose the B1F tab on the top left of the page.

Second, the accessible regions on Level B-1 of this shopping mall are mainly composed of a main corridor and several small passages and many shops. There are no ring paths so that there is always only one correct direction if a consumer wants to find a target shop. This makes it possible to evaluate the error rate in navigation. It should be noted that in most indoor environments, users can move with more than two directions (e.g., making a turn to left or right instead of moving forward or backward, or even wandering around an area). It is possible to let Canoe notify users to make a turn instead of moving “forward or backward”. Nonetheless, it is difficult for Canoe to decide which turn to make. Another choice is to let users make the decision by themselves. When the distance between the user and the destination decreases and then increases, there are two possibilities: the user has just passed the destination or the user should make a turn. If the destination is not nearby, the user should make a turn. If the user makes a wrong turn, the distance will continue to increase, and Canoe will notify the user to move backward.

Third, the performance of Canoe depends strongly on the complexity of the environments. It should be noted that within a complex in-building environment, it is very difficult to find the best path without a perfect floor plan. Suppose a “C”-shaped corridor if the user and the destination are located at different end-points. In this case, Canoe will point to the relative direction instead of the correct moving direction. The floor plan of Level B-1 is relatively simple like most real in-building shopping environments. Thus, consumers are able to find their destinations if they know relative directions. Our experiments are performed on this floor.

On Floor B-1, there are in total 71 different shops, where 55 of these shops have an AP with a unique Service Set Identifier (SSID). Notice that we do not use Media Access Control (MAC)-addresses (but only the SSIDs) to distinguish the APs. This is because we do not want to use any AP, which is publicly deployed by the building owner. The APs deployed by the shopping mall are always named “DeJiPlaza” or “DeJiPlaza-POS” which refers to the name or the points-of-sale of the building. By using the SSIDs, we choose only personally-deployed APs. Thus, the experiment results may offer insight to the performance of Canoe in other buildings, which may not have publicly-deployed APs.

Based on the RSS measurements from these APs, Canoe provides navigation service to both the shop-users and the consumer-users. A shop-user can use Canoe to guide consumers to his/her own shop, by uploading the RSS measurements he or she collects at the entrance of the shop; a consumer-user can use Canoe to find his/her target shop, by continuously collecting RSS measurements along his or her path and comparing these measurements with those published by the shop-user. In our experiments, since some shops are much smaller than others, we ignore these small shops. At last, we choose 64 observing locations, each of which is an entrance of a shop. Under the assumptions that 1/4, 1/3 or 1/2 of the shop-owners are willing to use Canoe, we have conducted three groups of experiments. The first group uses RSS measurements of $n_{s}=16$ shops (with two shops in between neighboring observing locations); the second group uses RSS measurements of $n_{s}=21$ shops (with three shops in between neighboring observing locations), while the third group uses RSS measurements of $n_{s}=32$ random shops (with fourshops in between neighboring observing locations).

Our experiments were performed in 14 different afternoons. In every afternoon, we used different mobile devices to collect the RSS measurements at each observing location, i.e., the entrance of each shop (which are re-collected every about two hours, since RSS value is dynamic). Then, we randomly selected a shop as the target shop. Consumers were asked to walk freely, and they did not know which shop was the target shop. By doing this, their familiarity of this shop or to this shopping mall could be omitted. At last, 30 long traces are collected (which are later divided into 200 short traces). Along the consumer’s path, Canoe tells whether he or she is approaching or leaving the target shop every three seconds. This answer is recorded and later compared with the correct answer.

We have evaluated the effectiveness of Canoe using the metrics of failure rate and error rate. A “failure” occurs if Canoe cannot compute a result because the information is insufficient for estimation (i.e., no result is generated by Canoe). An “error” occurs if Canoe computes a result that is wrong (i.e., Canoe generates a result “forward”, while the correct direction should be “backward” or vice versa). We do not count failures as errors. The failure rate is defined as failure/total, and the error rate is defined as error/(total−failure). The average failure rate and error rate of navigation of the total 200 traces are shown in Table 3, Table 4 and Table 5. If there are enough shop-users ($n_{s}=32$), the failure rate and error rate are very low. After the consumer-user performs nine to 11 RSS observations (i.e., after about half a minute), the error rate is below 10 %. Nonetheless, when there are not enough shop-users, it is difficult for Canoe to compute a correct result.

## 6. Related Work

Indoor localization and navigation have been extensively studied. In the following, we briefly summarize various techniques in this field, which are close to our study.

Indoor localization: A number of existing indoor localization systems has achieved a high position accuracy with dedicated hardware deployment, including localization based on ultra-high frequency (UHF) [14], ultra-wide band (UWB) [15], dedicated sensor or beacons [16] and others [17,18]. For instance, Tagoram [19], which uses radio frequency identification devices (RFID), can achieve mm-level accuracy.

To reduce the cost in deploying an indoor localization system, a number of research efforts has leveraged existing Wi-Fi infrastructure and introduced location fingerprinting based on RSS measurements of the Wi-Fi signals (e.g., radar [20]). The deployment of location fingerprinting systems is often divided into two phases: (i) an off-line phase, in which a site survey of the RSS from multiple APs are collected, and (ii) an on-line phase, in which a location can be computed based on the currently-observed RSS measurements.

The site survey in the off-line phase can be extremely time consuming and labor intensive. In the last few years, a number of investigations introduced crowd-sourcing-based systems [3,9,21,22,23], which require the users to continuously observe their RSS measurements and upload the data to the positioning server. These approaches do not require the site survey to be performed, and they do not require the map of the floor-plan. Nonetheless, additional incentive mechanisms are required to attract enough participants, as anyone uploading his or her observed RSS measurements needs to obtain a benefit, like positioning accuracy, but will definitely still incur privacy risks [24].

Unlike existing indoor localization systems, which require a site survey, knowledge of the layout or floor-plan, AP placement and power settings or cooperative users, Canoe uses only the RSS measurements of the positioning customer and the participating shops. It is worth noting that Canoe cannot provide accurate localization, but can provide precise navigation and does not require any deployment of localization services.

Indoor Navigation: Most existing indoor navigation systems should first localize the users [25], while in some approaches, users’ absolute locations are not necessary. For example, Escort [26] could record users’ movement patterns and mutual encounters, so as to help a person navigate to another person. Travi-Navi [27] is a self-deployable indoor navigation system, which records images and sensor readings along a guide’s path and are then used to direct followers. Unlike previous approaches, which require a guide to record various features along the path, and require the followers to move in exactly the same path as the guide, Canoe only requires the features at the customer and destination (RSS observations at the shops) and does not limit the paths of the followers.

## 7. Conclusions

In this paper, we have presented Canoe, a novel indoor navigation system, which does not require the deployment of localization services and prior knowledge of the environment. With Canoe, shop-owners can guide consumers to their shops by only publishing RSS measurements collected at the shop entrances, while the consumer can be guided to their target shop by continuously observing RSS measurements along their path. In Canoe, we have used maximum likelihood estimation and stochastic gradient descent optimization techniques to solve the dimension mismatch problem. In doing these, the locations of the shops and the consumer can be estimated accurately. We have also designed an early stopping strategy to deal with the overfitting issue, such that the iteration process is curtailed when the learned results best describe the real locations of observations. With a combination of a simulation study based on real data and live experiments on a real-world shopping mall environment, our experimental results show that Canoe is applicable and feasible in real-world indoor navigation environments.

## Figures and Tables

**Table 1:** Summary of notations.

Symbol	Meaning
A,A	AP, the set of all APs
$n_{s},n_{c}$	the number of locations where RSS are observed by the shops or by the consumer
$I^{\prime },I^{\prime \prime }$	matrix of RSS measurements observed by the shops or by the consumer
$I,\tilde{I}$	matrix of all RSS measurements ($I=I^{\prime }+I^{\prime \prime }$), matrix of computed RSS
$I,I_{j},I_{i,j}$	RSS observation, RSS measurement from the *j*-th AP, the *i*-th RSS measurement from the *j*-th AP
$D_{i,j}$	distance between the *i*-th observing location and the *j*-th AP
$P_{i},Q_{j}$	location of the *i*-th observation, location of the *j*-th AP

**Table 2:** Error rate in navigation after $n_{c}$ observations.

$n_{c}=2$	$n_{c}=3$	$n_{c}=4$	$n_{c}=5$
0.25	0.17	0.10	0.10

**Table 3:** Failure rate and error rate (16 shops).

$n_{s}=16$	$n_{c}=3$	$n_{c}=5$	$n_{c}=7$	$n_{c}=9$	$n_{c}=11$
Failure	0.66	0.59	0.42	0.44	0.37
Error	0.37	0.29	0.25	0.27	0.19

**Table 4:** Failure rate and error rate (21 shops).

$n_{s}=21$	$n_{c}=3$	$n_{c}=5$	$n_{c}=7$	$n_{c}=9$	$n_{c}=11$
Failure	0.55	0.40	0.31	0.21	0.19
Error	0.39	0.28	0.23	0.21	0.17

**Table 5:** Failure rate and error rate (32 shops).

$n_{s}=32$	$n_{c}=3$	$n_{c}=5$	$n_{c}=7$	$n_{c}=9$	$n_{c}=11$
Failure	0.38	0.20	0.11	0.07	0.03
Error	0.34	0.22	0.18	0.09	0.08

## References

[1] Lin J., Yu W., Zhang N., Yang X., Zhang H., Zhao W. (2017). A Survey on Internet of Things: Architecture, Enabling Technologies, Security and Privacy, and Applications. IEEE Int. Things J..

[2] Kaemarungsi K., Krishnamurthy P. Modeling of indoor positioning systems based on location fingerprinting. Proceedings of the 23rd IEEE Annual Joint Conference of the IEEE Computer and Communications Societies (INFOCOM).

[3] Chintalapudi K., Padmanabha Iyer A., Padmanabhan V.N. Indoor localization without the pain. Proceedings of the ACM 16th Annual International Conference on Mobile Computing and Networking.

[4] Sheng K., Gu Z., Mao X., Tian X., Wu W., Gan X., Wang X. The Collocation of Measurement Points in Large Open Indoor Environment. Proceedings of the 2015 IEEE Conference on Computer Communications.

[5] Beder C., Klepal M. Fingerprinting based localisation revisited: A rigorous approach for comparing RSSI measurements coping with missed access points and differing antenna attenuations. Proceedings of the 2012 IEEE International Conference on Indoor Positioning and Indoor Navigation.

[6] Kushki A., Plataniotis K.N., Venetsanopoulos A.N. (2007). Kernel-based positioning in wireless local area networks. IEEE Trans. Mob. Comput..

[7] Gansemer S., Großmann U., Hakobyan S. Rssi-based euclidean distance algorithm for indoor positioning adapted for the use in dynamically changing wlan environments and multi-level buildings. Proceedings of the International Journal of Indoor Positioning and Indoor Navigation.

[8] Google Indoor Maps availability. https://support.google.com/gmm/answer/1685827?hl=en.

[9] Yang Z., Wu C., Liu Y. Locating in fingerprint space: Wireless indoor localization with little human intervention. Proceedings of the 18th ACM Annual International Conference on Mobile Computing and Networking.

[10] Wu C., Yang Z., Liu Y., Xi W. (2013). WILL: Wireless indoor localization without site survey. IEEE Trans. Parallel Distrib. Syst..

[11] Kaemarungsi K., Krishnamurthy P. Properties of indoor received signal strength for WLAN location fingerprinting. Proceedings of the ACM International Conference on Mobile and Ubiquitous Systems: Networking and Services.

[12] A Community Resource for Archiving Wireless Data At Dartmouth. http://crawdad.org/mannheim/compass.

[13] DeJi Floor-plan availability. http://m.dejiplaza.com/vmall/html/shoppingguide.jsp.

[14] Werb J., Lanzl C. (1998). Designing a positioning system for finding things and people indoors. IEEE Spectr..

[15] Fontana R.J., Richley E., Barney J. Commercialization of an ultra wideband precision asset location system. Proceedings of the 2003 IEEE Conference on Ultra Wideband Systems and Technologies.

[16] Priyantha N.B., Chakraborty A., Balakrishnan H. The cricket location-support system. Proceedings of the 6th ACM Annual International Conference on Mobile Computing and Networking.

[17] Wang J., Chen Y., Fu X., Wang J., Yu W., Zhang N. 3DLoc: Three Dimensional Wireless Localization Toolkit. Proceedings of the IEEE International Conference on Distributed Computing Systems.

[18] Fu X., Zhang N., Pingley A., Yu W., Wang J., Zhao W. (2012). The Digital Marauder’s Map: A WiFi Forensic Positioning Tool. IEEE Trans. Mob. Comput..

[19] Yang L., Chen Y., Li X.Y., Xiao C., Li M., Liu Y. Tagoram: Real-time tracking of mobile RFID tags to high precision using COTS devices. Proceedings of the 20th ACM Annual International Conference on Mobile Computing and Networking.

[20] Bahl P., Padmanabhan V.N. RADAR: An in-building RF-based user location and tracking system. Proceedings of the 19th IEEE Annual Joint Conference of the IEEE Computer and Communications Societies.

[21] Rai A., Chintalapudi K.K., Padmanabhan V.N., Sen R. Zee: Zero-effort crowdsourcing for indoor localization. Proceedings of the 18th ACM Annual International Conference on Mobile Computing and Networking.

[22] Wang H., Sen S., Elgohary A., Farid M., Youssef M., Choudhury R.R. No need to war-drive: Unsupervised indoor localization. Proceedings of the 10th ACM International Conference on Mobile Systems, Applications, and Services.

[23] Purohit A., Sun Z., Pan S., Zhang P. SugarTrail: Indoor navigation in retail environments without surveys and maps. Proceedings of the 2013 10th Annual IEEE Communications Society Conference on Sensor, Mesh and Ad Hoc Communications and Networks.

[24] Zhao C., Yang X., Yu W., Yao X., Lin J., Li X. (2007). Cheating-Resilient Incentive Mechanism for Mobile Crowdsensing Systems. arXiv.

[25] Fallah N., Apostolopoulos I., Bekris K., Folmer E. (2013). Indoor human navigation systems: A survey. Int. J. Interact. Comput..

[26] Constandache I., Bao X., Azizyan M., Choudhury R.R. Did you see bob?: Human localization using mobile phones. Proceedings of the 16th ACM Annual International Conference on Mobile Computing and Networking.

[27] Zheng Y., Shen G., Li L., Zhao C., Li M., Zhao F. Travi-navi: Self-deployable indoor navigation system. Proceedings of the 20th ACM Annual International Conference on Mobile Computing and Networking.

